# Yb-Doped CuY modulates Cu electronic structures for efficient oxidation of anisole to guaiacol†

**DOI:** 10.1039/d5ra00463b

**Published:** 2025-04-01

**Authors:** Hanghang Zhang, Gang Xu

**Affiliations:** a ^a^, Polytechnic Institute, Zhejiang University Hangzhou 310015 China; b College of Chemical and Biological Engineering, Zhejiang University Hangzhou 310058 China xugang_1030@zju.edu.cn

## Abstract

Copper-based catalysts are widely employed in the catalytic conversion of aromatic compounds to phenolic derivatives. However, single-atom copper catalysts often exhibit limited reactivity and stability. In this study, we addressed these limitations by loading Cu onto NaY zeolite *via* ion exchange to synthesize a CuY catalyst, followed by Yb modification. By optimizing the Yb content, we significantly enhanced the anisole conversion rate and guaiacol yield. Catalyst characterization revealed that Yb species modulate the electronic structure of copper, favoring the formation of abundant Cu^+^ species. This electronic modification promotes the catalytic generation of hydroxyl radicals (˙OH) from hydrogen peroxide, thereby accelerating the reaction kinetics. Furthermore, Yb incorporation into CuY induced the formation of oxygen vacancies, which improved hydroxyl radical adsorption and facilitated the generation of metal-oxo species. These synergistic effects collectively increased the reaction's overall conversion efficiency. Under optimized reaction conditions, the Yb/CuY catalyst achieved an anisole conversion of 56.2% and an *ortho*-selectivity of 74.6%, clearly outperforming previous reports.

## Abstract 2

Yb-Doped CuY catalysts enhance the electronic structure of copper for efficient hydrogen peroxide oxidation of anisole to guaiacol, achieving improved conversion rates and selectivity.

## Graphical abstract

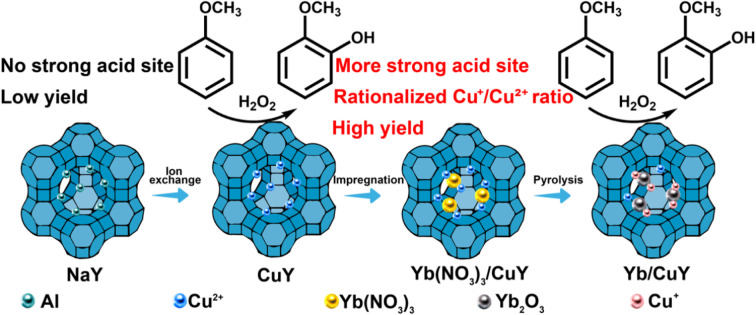

## 1 Introduction

Guaiacol is an important natural phenolic compound widely used in the pharmaceutical, fragrance, and food industries.^1–5^ The one-step oxidation of anisole to guaiacol using hydrogen peroxide has the advantages of simplicity, environmental friendliness, few by-products, and high selectivity.^6–9^ However, the reaction of directly using hydrogen peroxide to oxidize anisole to guaiacol shows almost no catalytic activity.^10–13^ Therefore, current research primarily focuses on developing efficient catalysts.^14–17^ To this end, researchers have combined metal catalysts (such as iron and copper) with carbon materials, zeolites, and metal oxides to construct heterogeneous catalyst systems,^18–22^ which effectively promote the oxidation of anisole to guaiacol. For example, Zhu *et al.*^23^ developed a CNT@FcPOP catalyst in which CNTs reduce Fe^3+^ to Fe^2+^, which then reacts with H_2_O_2_ to produce hydroxyl radicals that attack the aromatic ring, yielding phenol. The macroporous and mesoporous structure of CNTs enhances mass transfer between reactants and products, promoting the reaction and achieving a phenol yield of 25.3%. Zeng *et al.*^24^ showed that a bimetallic FeCo nanoalloy can directly hydroxylate benzene to phenol. Qi *et al.*^25^ used a tetraaminobenzimidazole copper perchlorate complex (Cu(L_1_)(ClO_4_)_2_, where L_1_ is tetraaminobenzimidazole) for benzene hydroxylation to phenol. In this system, Cu^2+^ attacks the benzene ring *via* electrophilic substitution, achieving a benzene conversion rate of 22.4%. Zhang *et al.*^26^ loaded a copper–palladium bimetal onto graphitic carbon nitride for photocatalytic benzene hydroxylation, achieving a conversion rate of 98.1% and a phenol selectivity of 89.6%. Wu *et al.*^27^ loaded an Fe–Cu bimetal onto SBA-15 zeolite for the oxidation of benzene to phenol using hydrogen peroxide. The addition of Cu to Fe-SBA-15 prevented the over-oxidation of phenol, improving selectivity and achieving a benzene conversion of 35.5% and a phenol selectivity of 89.8%. Basyach *et al.*^15^ loaded nickel onto g-C_3_N_4_ for benzene hydroxylation to phenol, achieving a conversion rate of 98.5% and a selectivity of 82.7%. Mizuno *et al.*^18^ synthesized γ-[PW_10_O_38_V_2_(μ-OH)_2_]^3−^ for the direct hydroxylation of anisole with hydrogen peroxide, achieving a conversion rate of 14%. P. Mukherjee *et al.*^28^ synthesized TS-1 for catalyzing the hydrogen peroxide oxidation of anisole, achieving a conversion rate of 60% (conversion is based on the number of moles of H_2_O_2_ taken with respect to anisole) and a selectivity of 65% for *p*-hydroxy-anisole. Pengyu Zhao *et al.*^29^ synthesized TS-1-C_INJ_ for catalyzing the hydrogen peroxide oxidation of anisole, achieving a conversion rate of 14.2%.

Although progress has been made in catalyst design, several limitations still exist. Catalysts often suffer from poor stability, making them prone to deactivation or degradation, which negatively impacts their performance. Additionally, while some catalysts achieve high conversion rates, their selectivity is frequently limited, leading to the formation of by-products. Moreover, some catalytic systems require stringent reaction conditions, restricting their broader applicability in industrial processes. Therefore, improving the stability, selectivity, and catalytic performance of catalysts is essential for effective design. Hydroxyl radicals, which are key reactive intermediates, easily react with hydrogen peroxide to form oxygen and water. Consequently, enhancing the efficiency of catalyst-mediated hydroxyl radical generation from hydrogen peroxide and improving the catalyst's ability to adsorb these radicals are critical for the reaction.

Therefore, we propose a new strategy by doping ytterbium (Yb) atoms into divalent copper catalysts, which alters the electronic structure of Cu^2+^ and promotes the partial conversion of Cu^2+^ to Cu^+^. At the same time, doping Yb atoms increases the oxygen vacancy content in the catalyst, thereby enhancing its catalytic activity. Specifically, this strategy achieves the following objectives: Cu^+^ can directly react with hydrogen peroxide to generate hydroxyl radicals. The increase in oxygen vacancies enhances the catalyst's ability to adsorb ˙OH radicals, promotes the generation of more reactive oxygen species, and improves the conversion rate. Additionally, the increase in strong acidic sites in the Yb-doped copper catalyst facilitates proton transfer, accelerating the reaction. This strategy enhances the conversion rate of anisole and optimizes overall reaction performance, fostering the further development of this catalytic system. Under optimized reaction conditions, the Yb/CuY catalyst achieved an anisole conversion of 56.2% and an *ortho*-selectivity of 74.6%, clearly outperforming previous reports. In addition, the catalyst exhibited excellent catalytic performance for various anisole derivatives (Table 1).

**Table 1:** Data showing the performances of different catalysts

Catalyst	Reactant	Yield (guaiacol)
Iron(ii) complex^17^	Anisole	10%
[Fe^II^(TPEN)]^2+^ (ref. 18)	Anisole	0.56%
γ-[PW_10_O_38_V_2_(μ-OH)_2_]^3−^ (ref. 19)	Anisole	14%
V–Si–ZSM-22 (ref. 20)	Anisole	14.1%
TS-1 (ref. 28)	Anisole	39%
TS-1-C_INJ_^29^	Anisole	9.8%
7% Yb/CuY (our work)	Anisole	41.9%

## 2 Experimental

### 2.1 Materials

Copper(ii) nitrate trihydrate (Cu(NO_3_)_2_·3H_2_O), zirconium nitrate, scandium nitrate ethanol, acetonitrile (CH_3_CN), sodium hydroxide (NaOH) were purchased from Aladdin Chemical Reagent Company. Sodium aluminate (NaAlO_2_), ytterbium(iii) nitrate pentahydrate (Yb(NO_3_)_3_·5H_2_O, 99.99%), nickel(ii) chloride, cobalt nitrate, cerium nitrate, aluminum nitrate, platinum(ii) chloride, palladium chloride, anisole (C_6_H_5_OCH_3_), *p*-fluoroanisole, *p*-chloroanisole, *p*-bromoanisole were purchased from Shanghai Meryer Chemical Technology Co., Ltd. Ludox HS-40 (40% silica gel) was acquired from Sigma-Aldrich. Hydrogen peroxide (H_2_O_2_, 30wt%) was acquired from Sinopharm Chemical Reagents Company. All chemicals were used as received without further purification. Cu/C was purchased from Scientific Materials Station, and HZSM-5 was purchased from Nankai University Catalyst Factory.

### 2.2 Catalyst preparation

#### 2.2.1 Synthesis of NaY

The starting materials NaOH, NaAlO_2_, deionized water, and Ludox HS-40 (40% silica gel) were mixed in a molar ratio of 6 : 1 : 6 : 130. The detailed synthesis procedure is as follows: NaOH was dissolved in deionized water with stirring until homogeneous, followed by the slow addition of NaAlO_2_ with continuous stirring. Ludox HS-40 was then added, and the mixture was aged for 100 hours. After aging, the mixture was heated at 100 °C for 48 hours for crystallization. The resulting solid was collected by centrifugation, washed three times with deionized water, and dried in an oven at 100 °C. The dried solid was calcined in a muffle furnace at 550 °C for 3 hours at a heating rate of 5 °C min^−1^ to obtain the NaY zeolite.

#### 2.2.2 Synthesis of CuY

2.0 g of NaY zeolite was placed in a 250 mL beaker, and 10 mL of water was added, followed by ultrasonic dispersion. Subsequently, 90 mL of 0.1 M copper nitrate aqueous solution was added to the mixture, stirred until homogeneous, and ultrasonicated for 30 minutes. The mixture was then placed in a water bath and heated to 90 °C with stirring for 2 hours. The resulting solid was collected by centrifugation, washed three times with deionized water, and dried in an oven at 100 °C. The dried solid was calcined in a muffle furnace at 550 °C for 3 hours at a heating rate of 5 °C min^−1^ to obtain the CuY zeolite.

#### 2.2.3 Synthesis of FeY

2.0 g of NaY zeolite was placed in a 250 mL beaker, and 10 mL of water was added, followed by ultrasonic dispersion. Subsequently, 90 mL of 0.1 M iron nitrate aqueous solution was added to the mixture, stirred until homogeneous, and ultrasonicated for 30 minutes. The mixture was then placed in a water bath and heated to 90 °C with stirring for 2 hours. The resulting solid was collected by centrifugation, washed three times with deionized water, and dried in an oven at 100 °C. The dried solid was calcined in a muffle furnace at 550 °C for 3 hours at a heating rate of 5 °C min^−1^ to obtain the FeY zeolite.

#### 2.2.4 Synthesis of YbY

The synthesis procedure was similar to that of CuY, except that the copper nitrate solution was replaced with ytterbium nitrate solution.

#### 2.2.5 Synthesis of Yb/CuY

A simple impregnation method was used to synthesize *x* Yb/CuY zeolites with different Yb loadings (*x* represents the mass percentage of Yb in CuY), including 1%, 2%, 4%, 6%, 7%, and 8%. For the preparation of 2% Yb/CuY, 1.0 g of CuY zeolite was placed in a 250 mL beaker, and 50 mL of water was added for ultrasonic dispersion. Then, 51 mg of Yb(NO_3_)_3_·5H_2_O was dissolved in 20 mL of water and added to the above mixture, which was stirred at room temperature for 24 hours. The resulting solid was separated by centrifugation, washed three times with deionized water, and dried in an oven at 100 °C. The dried solid was calcined in a muffle furnace at 550 °C for 3 hours at a heating rate of 5 °C min^−1^ to obtain the 2% Yb/CuY catalyst. Following the same procedure but using 26 mg, 104 mg, 156 mg, 182 mg, and 208 mg of Yb(NO_3_)_3_·5H_2_O, 1% Yb/CuY, 4% Yb/CuY, 6% Yb/CuY, 7% Yb/CuY, and 8% Yb/CuY catalysts were synthesized.

#### 2.2.6 Synthesis of YbCu/NaY

A simple impregnation method was used to synthesize YbCu/NaY. 1 g of NaY zeolite was placed in a 250 mL beaker, and 50 mL of water was added for ultrasonic dispersion. Then, 151 mg of Cu(NO_3_)_2_·3H_2_O and 182 mg of Yb(NO_3_)_3_·5H_2_O were dissolved in 20 mL of water and added to the mixture, which was stirred at room temperature for 24 hours. The resulting solid was separated by centrifugation, washed three times with deionized water, and dried in an oven at 100 °C. The dried solid was calcined in a muffle furnace at 550 °C for 3 hours at a heating rate of 5 °C min^−1^ to obtain the YbCu/NaY catalyst.

#### 2.2.7 Synthesis of Cu/YbY

A simple impregnation method was used to synthesize the Cu/YbY catalyst. 1.0 g of YbY zeolite was placed in a 250 mL beaker, and 50 mL of water was added for ultrasonic dispersion. Then, 151 mg of Cu(NO_3_)_2_·3H_2_O was dissolved in 20 mL of water and added to the mixture, which was stirred at room temperature for 24 hours. The resulting solid was separated by centrifugation, washed three times with deionized water, and dried in an oven at 100 °C. The dried solid was calcined in a muffle furnace at 550 °C for 3 hours at a heating rate of 5 °C min^−1^ to obtain the Cu/YbY catalyst.

### 2.3 Catalyst characterization

X-ray diffraction (XRD) patterns were collected using a Bruker D8 Advance diffractometer with Cu Kα radiation, a scanning rate of 2° min^−1^, and a scanning range of 5° to 90°. The voltage and current were set at 40 kV and 40 mA, respectively. Ammonia temperature-programmed desorption (NH_3_-TPD) experiments were performed on a MicrotracBET BELCAT II instrument. The samples were first heated to 500 °C in an argon flow (30 mL min^−1^) and kept at this temperature for 1 hour, then cooled to 100 °C. A mixture of 10 vol% NH_3_ in Ar was introduced at a flow rate of 30 mL min^−1^ for adsorption for 1 hour. Subsequently, the sample was purged with argon (30 mL min^−1^) at 100 °C for 1 hour to remove physically adsorbed NH_3_. Finally, the sample was heated to 900 °C at a rate of 10 °C min^−1^, and the desorption was recorded. The textural parameters of the catalysts were determined by N_2_ sorption isotherms using a Micromeritics ASAP 2460 specific surface area and porous physical adsorption analyzer. Thermogravimetric curves (TG) were performed on an Q5000 Simultaneous DSC-TGA in flowing air with a heating rate of 10 °C min^−1^. Fourier-transform infrared (FTIR) spectroscopy was conducted using a Bruker INVENIO spectrometer. The absorbance of the solution was measured using an Agilent Cary 7000 Universal Measurement Spectrophotometer (UMS). Scanning electron microscopy (SEM) images were obtained using a JSM-7800F Prime instrument. Transmission electron microscopy (TEM) and high-resolution TEM (HRTEM) images were acquired using an FEI Talos F200i microscope operating at 200 kV. The catalyst composition was determined using inductively coupled plasma mass spectrometry (ICP-MS) with an Agilent 7900 instrument. *In situ* X-ray photoelectron spectroscopy (XPS) was performed using a Thermo ESCALAB 250 XI spectrometer. EPR was tested using a Bruker A300. Raman spectroscopy was measured using an inVia-reflex. *In situ* infrared spectroscopy was measured using an INVENIO.

### 2.4 Catalytic experiment

The catalytic oxidation of anisole was carried out in a 10 mL glass reaction tube. The typical experimental procedure is as follows: 10 mg of the prepared catalyst was placed in a 10 mL glass reaction tube, and solvent (a mixture of acetonitrile and water in a volume ratio of 1 mL : 1 mL) was added, followed by ultrasonic dispersion. Then, 90 mg of anisole was added, and the mixture was heated to 80 °C. Subsequently, 200 μL of hydrogen peroxide (30 wt%) was added, and the reaction was conducted for 30 minutes. After the reaction, the mixture was cooled to room temperature, and unreacted hydrogen peroxide was quenched by adding sodium thiosulfate. Then, 60 μL of ethylbenzene was added as an internal standard. The mixture was extracted with 20 mL of ethyl acetate and centrifuged. The resulting solution was analyzed by gas chromatography (GC, 9790plus) equipped with a flame ionization detector and an RB-5 capillary column (0.32 mm). The catalyst was separated by centrifugation, washed three times with water and three times with ethanol, and dried in an oven at 100 °C. The dried solid was calcined in a muffle furnace at 550 °C for 3 hours at a heating rate of 5 °C min^−1^ to evaluate the reusability of the catalyst. Other experiments differed in the type and ratio of solvents, the volume of hydrogen peroxide (100 μL, 150 μL, 200 μL, 250 μL, 300 μL), the reaction temperature (50 °C, 60 °C, 70 °C, 80 °C, 90 °C), and the use of different catalysts for comparison experiments.

The conversion of anisole and the selectivity of guaiacol were calculated according to the following formulas:
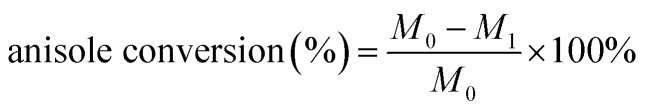

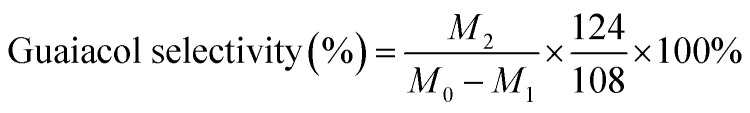
*M*_0_ and *M*_1_ represent the mass of anisole added and the remaining mass after the reaction, respectively. *M*_2_ represents the mass of guaiacol produced after the reaction.

## 3 Results and discussion

### 3.1 Characterization results

**Fig. 1:**
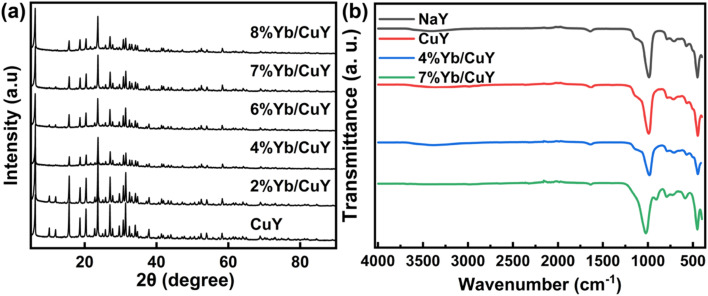
(a) Structural characterizations. XRD patterns of CuY, 2% Yb/CuY, 4% Yb/CuY, 6% Yb/CuY, 7% Yb/CuY and 8% Yb/CuY. (b) FTIR spectra of NaY, CuY, 4% Yb/CuY and 7% Yb/CuY.

CuY and YbY catalysts were prepared by ion exchange, and a series of Yb/CuY zeolites with varying Yb loadings were synthesized *via* impregnation. Fig. 1(a) presents the XRD patterns of CuY, 2% Yb/CuY, 4% Yb/CuY, 6% Yb/CuY, 7% Yb/CuY, and 8% Yb/CuY samples, showing diffraction peaks at 7.1°, 10.0°, 12.5°, 15.0°, 23.0°, 27.0°, and 30.0°, which correspond to the NaY zeolite. The XRD patterns of CuY, YbY, and Yb/CuY zeolites are similar, all retaining the characteristic diffraction peaks of NaY zeolite, though the peak intensities have changed. This indicates that while the pore structure was partially damaged during the zeolite modification, the original lattice structure of the NaY zeolite remained intact.

Fig. 1(b) presents the Fourier-transform infrared (FTIR) spectra of NaY, CuY, 4% Yb/CuY, and 7% Yb/CuY. From the figure, it can be observed that all four types of zeolites exhibit internal tetrahedral asymmetric and symmetric stretching vibration peaks (1141 cm^−1^ and 795 cm^−1^), external tetrahedral asymmetric and symmetric stretching vibration peaks (1020 cm^−1^ and 718 cm^−1^), and the stretching vibration absorption peak of the external linkage of double six-membered rings (460 cm^−1^), consistent with the infrared data reported for Y-type zeolite structures in the literature. This indicates that the zeolite framework of all four catalysts remained structurally intact.

**Fig. 2:**
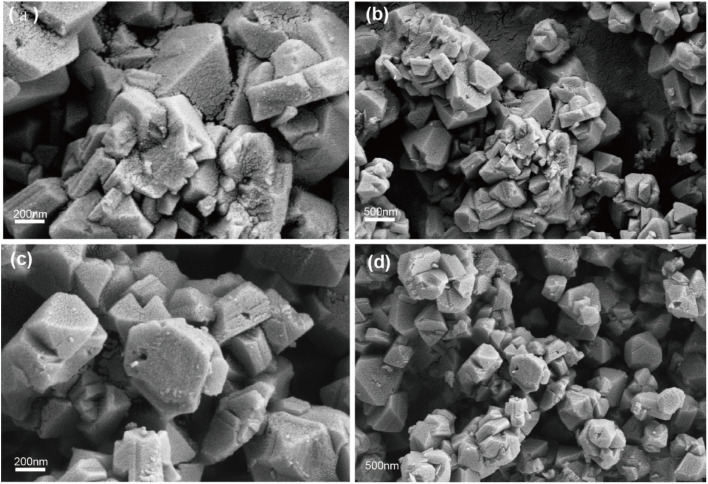
(a) and (b) SEM images of CuY. (c) and (d) SEM image of 7% Yb/CuY.

The morphology of the catalyst was directly observed using electron microscopy. Fig. 2(a), at a magnification of 200 nm, and Fig. 2(b), at 500 nm, show SEM images of the CuY zeolite. The average particle size of the CuY zeolite is clearly between 200 and 500 nm, exhibiting a regular octahedral morphology typical of the FAU-type zeolite structure. The particle surfaces are slightly rough, possibly due to partial lattice collapse of the NaY zeolite during the metal ion exchange process, resulting in some damage. However, the crystal edges remain distinct, indicating good crystallinity. There is some degree of particle agglomeration, but the overall dispersion is favorable, displaying good morphological characteristics. Fig. 2(c), at a magnification of 200 nm, and Fig. 2(d), at 500 nm, show SEM images of the 7% Yb/CuY zeolite.

**Fig. 3:**
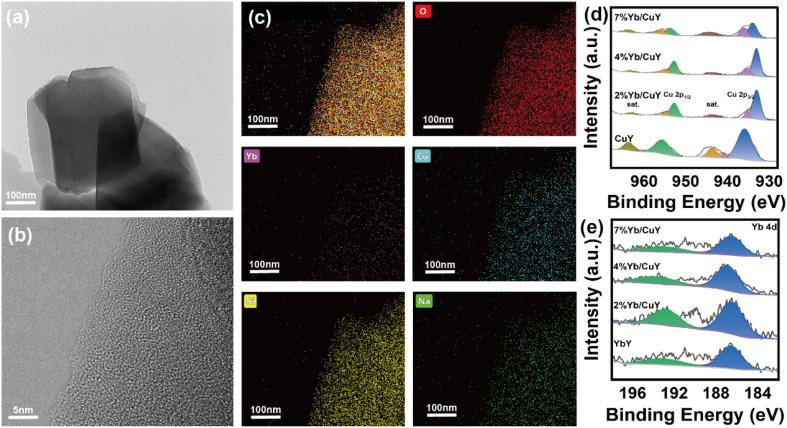
(a) TEM and (b) HRTEM images of 7% Yb/CuY; (c) EDS elemental mapping of 7% Yb/CuY (d) XPS spectra of Cu 2p of CuY, 2% Yb/CuY, 4% Yb/CuY and 7% Yb/CuY (e) XPS spectra of Yb 4d of CuY, 2% Yb/CuY, 4% Yb/CuY and 7% Yb/CuY.

As shown in Fig. 3(c), Yb and Cu are uniformly distributed within the zeolite, and the stable zeolite framework effectively prevents the leaching of Yb and Cu. X-ray photoelectron spectroscopy (XPS) was used to analyze the surface chemical composition and oxidation states of the elements. The Cu 2p XPS spectra are presented in Fig. 3(d), where the peaks at 933.3 eV and 953.3 eV are attributed to Cu^2+^, corresponding to Cu 2p_3/2_ and Cu 2p_1/2_, respectively. Satellite peaks are observed at 962.3 eV and in the range of 940.6–943.6 eV, indicating that the primary copper species in the CuY catalyst is Cu^2+^. The presence of Cu^2+^ species is also observed in the 2% Yb/CuY, 4% Yb/CuY, and 7% Yb/CuY catalysts. The peak at 933 eV is attributed to Cu^+^. Fig. 3(d) shows that Cu^+^ species are present in the 2% Yb/CuY, 4% Yb/CuY, and 7% Yb/CuY catalysts, while Cu^+^ is not found in the CuY catalyst, indicating that the introduction of Yb into the CuY catalyst results in the partial conversion of Cu^2+^ to Cu^+^. As shown in Table S1,† as Yb loading increases, the Cu^+^ : Cu^2+^ ratio gradually decreases, and the catalytic performance improves, indicating that Cu^+^ plays a key role in enhancing catalytic efficiency. However, an excessively high Cu^+^ ratio is not favorable for the reaction. The catalytic effect is optimal when the Cu^+^ : Cu^2+^ ratio reaches an appropriate balance, suggesting a synergistic effect between Cu^+^ and Cu^2+^ species in this catalytic system.

**Fig. 4:**
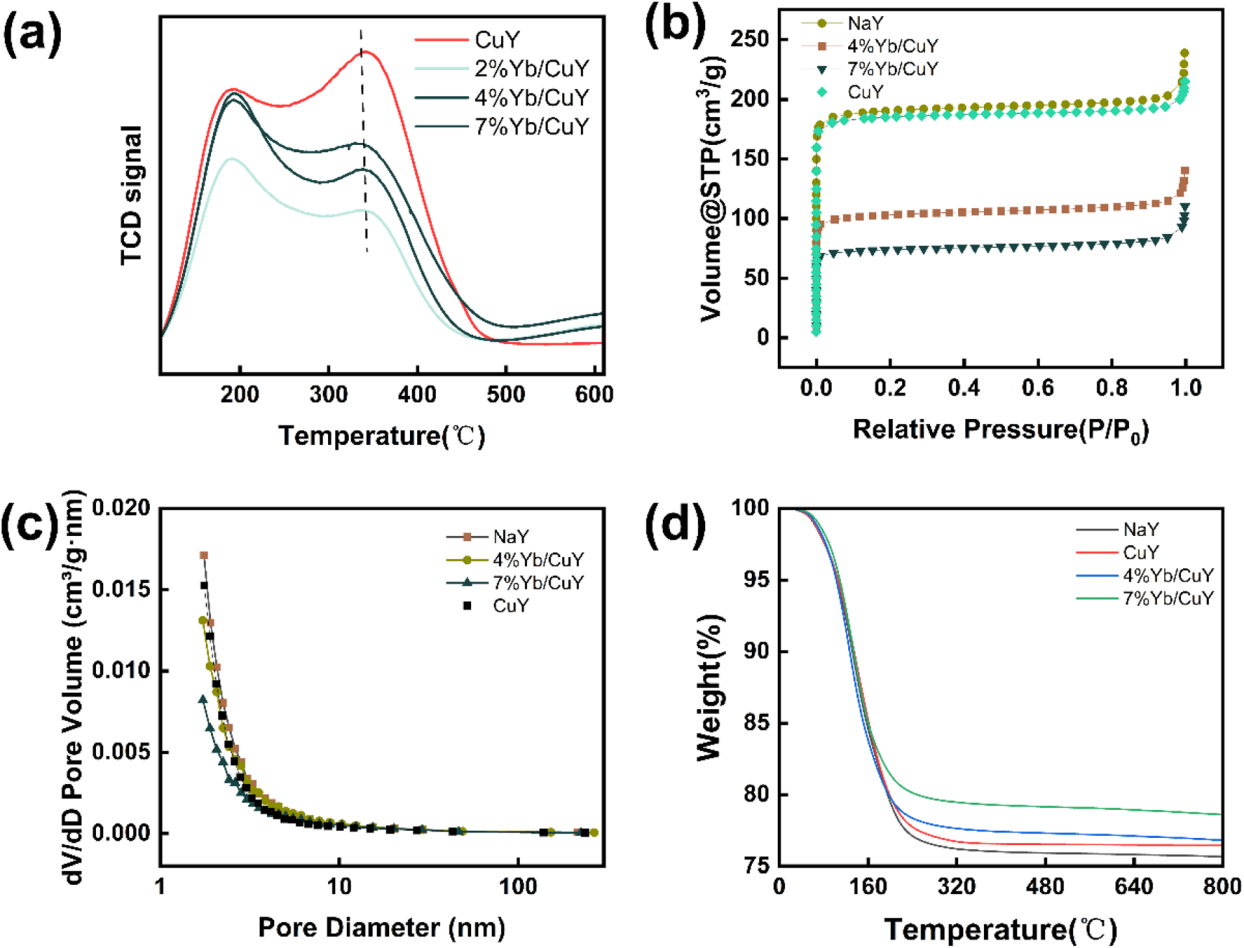
(a) TPD of ammonia of CuY, 2% Yb/CuY, 4% Yb/CuY and 7% Yb/CuY. (b) and (c) nitrogen adsorption and desorption of NaY, CuY, 4% Yb/CuY and 7% Yb/CuY (d) desorption thermogravimetric (TG) curves of NaY, CuY, 4% Yb/CuY and 7% Yb/CuY.

The changes in the acidity of the zeolite catalysts were observed through ammonia temperature-programmed desorption (NH_3_-TPD). The characteristic desorption curves of the catalysts are shown in Fig. 4(a). The desorption curves of the four catalysts-CuY, 2% Yb/CuY, 4% Yb/CuY, and 7% Yb/CuY-are similar, indicating minor differences in the acidic sites among these catalysts. Desorption peaks for all four catalysts appeared in the range of 100–600 °C. Typically, peaks below 200 °C correspond to weak acid centers, those between 200–400 °C correspond to medium-strong acid sites, and those above 400 °C represent strong acid sites. As shown in Fig. 4(a), all four catalysts exhibit weak and medium-strong acid centers, but only the Yb-modified catalysts show the presence of strong acid sites, which are absent in the CuY catalyst. This indicates that increasing Yb loading helps enhance the strong acid sites in the catalyst.

Quantitative analysis results of the desorbed NH_3_ using the Gaussian distribution method are presented in Table S2.† The number of strong acid centers for the four catalysts follows the order: 7% Yb/CuY > 4% Yb/CuY > 2% Yb/CuY. This suggests that the quantity of strong acid sites is positively correlated with catalytic efficiency, indicating that an increase in strong acid sites is beneficial for the stability of hydrogen peroxide and facilitates electron transfer, thereby enhancing reaction efficiency. With increasing Yb loading, the number of weak acid sites shows no significant difference, while the variation in medium-strong acid sites is considerable. However, there is no clear correlation between the medium-strong acid sites and substrate conversion, whereas a positive correlation is observed between the number of strong acid sites and catalytic efficiency, suggesting that strong acid sites are the key factor influencing catalytic performance.

Fig. 4(b) and (c) illustrate the changes in the surface area and pore volume of NaY, CuY, 4% Yb/CuY, and 7% Yb/CuY catalysts. As shown in Table S3,† the specific surface area and pore volume of the catalysts follow the order: NaY > CuY > 4% Yb/CuY > 7% Yb/CuY. After Cu^2+^ ion exchange, the changes in the specific surface area and pore volume of the zeolite are minor. However, after incorporating ytterbium, the specific surface area and pore volume of the zeolite decrease significantly. Combined with the XRD results, it can be concluded that the zeolite structure remains largely intact after Yb doping, suggesting that Yb doping may block some of the micropores, resulting in a decrease in surface area and pore volume. This reduction could explain the decline in catalytic performance observed at high Yb loading.

Fig. 4(d) presents the thermogravimetric (TG) curves of NaY, CuY, 4% Yb/CuY, and 7% Yb/CuY zeolites in an oxygen desorption atmosphere. As shown in the figure, significant weight loss occurs between 100–200 °C, primarily due to the evaporation of physically adsorbed water. The water adsorption capacity of the four zeolites follows the order: NaY > CuY > 4% Yb/CuY > 7% Yb/CuY. In the 200–400 °C range, there is a minor weight loss for all four zeolites, mainly attributed to the desorption of crystallized water, indicating a low content of crystallized water in these zeolites. In the 400–800 °C range, the TG curves gradually level off, suggesting that most volatile components have been removed and that all four zeolites exhibit good thermal stability.

### 3.2 Catalytic performance

#### 3.2.1 Effect of different catalysts

**Fig. 5:**
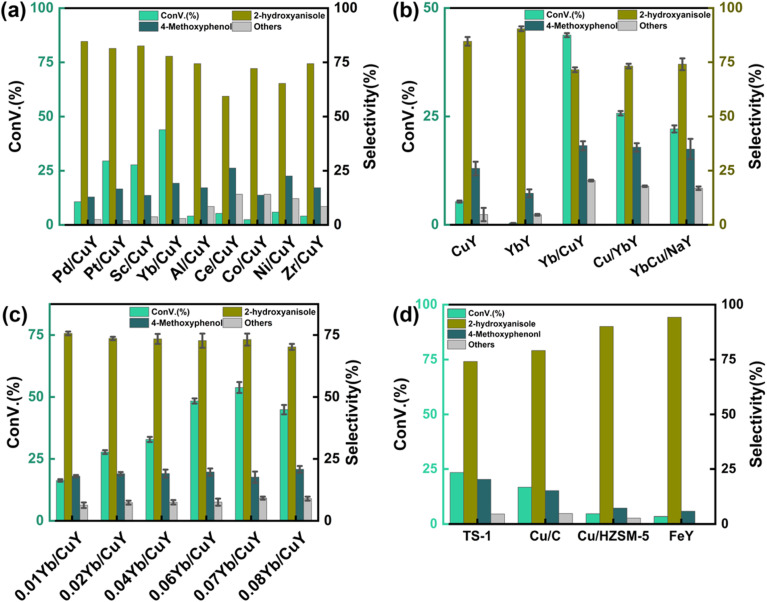
(a) Effect of different metal promoters; (b) effect of different loading methods; (c) effect of different Yb mass fractions. (d) Compared with other catalysts. Reaction conditions: anisole (90 mg), hydrogen peroxide (250 μL), catalyst (1 mg), solvent (5 mL each of acetonitrile and water), 80 °C, 30 min.

The catalytic effect of the CuY catalyst alone was limited, with an anisole conversion rate of only 5.6%. Different metals (Pd, Pt, Sc, Yb, Al, Ce, Co, Ni, Zr) were introduced into CuY as secondary metal promoters using the impregnation method to investigate their influence on the reaction. As shown in Fig. 5(a), the introduction of ytterbium had the greatest effect on the reaction. The Yb/CuY catalyst achieved an anisole conversion rate exceeding 40%, with guaiacol selectivity surpassing 70%. X-ray photoelectron spectroscopy (XPS) analysis showed that the introduction of ytterbium significantly increased the content of Cu^+^ species in the catalyst, which reacted with H_2_O_2_ to generate more hydroxyl radicals (˙OH) during the reaction, thereby favoring the hydroxylation and significantly improving the conversion rate.

Fig. 5(b) shows the effect of different loading methods on the reaction, primarily comparing the catalytic effects of Cu/YbY, YbCu/NaY, and Yb/CuY. The Cu/YbY catalyst was synthesized by ion-exchanging NaY with ytterbium ions to form YbY, followed by loading copper ions onto YbY through a simple impregnation method. The YbCu/NaY catalyst was prepared by first mixing ytterbium and copper nitrates into a solution and then loading it onto NaY *via* impregnation. The Yb/CuY catalyst was synthesized by ion-exchanging NaY with copper ions to form CuY, followed by loading ytterbium ions onto CuY through impregnation. As shown in Fig. 5(b), the Yb/CuY catalyst demonstrated the highest improvement in conversion rate. This may be due to the ion exchange of ytterbium ions with CuY, which potentially reduces the copper ion loading, whereas loading ytterbium and copper together onto NaY results in varied deposition positions for Yb and Cu. After confirming that Yb/CuY provided the highest improvement in conversion rate, the effect of different Yb loadings on the reaction was examined using 1%, 2%, 4%, 6%, 7%, and 8% Yb/CuY catalysts. Fig. 5(c) shows that at a Yb mass fraction of 7%, the conversion rate reached its peak, exceeding 50%, with guaiacol selectivity remaining above 70%.

According to the ammonia temperature-programmed desorption (NH_3_-TPD) characterization results, the desorption temperature of ammonia for the 7% Yb/CuY catalyst was the highest at 347.4 °C, and this catalyst also had the highest number of acidic sites. The increase in acidic sites is beneficial for stabilizing hydrogen peroxide, which in turn improves the conversion rate. ICP-OES results, shown in Table S4,† indicate that an increase in ytterbium loading leads to the loss of copper ions, which are crucial for the catalytic reaction. This loss of copper ions reduces the catalytic activity of the catalyst. Based on these experimental results, the 7% Yb/CuY catalyst demonstrated the best catalytic performance.

The reaction of hydrogen peroxide oxidation of anisole to guaiacol was catalyzed by TS-1, Cu/C, Cu/HZSM-5, and FeY catalysts. As shown in Fig. 5(d), TS-1 and Cu/C exhibited relatively good catalytic performance, but the conversion rate remained below 25%. In contrast, Cu/HZSM-5 and FeY showed poor performance. Among them, Yb/CuY demonstrated the best catalytic activity.

#### 3.2.2 Effect of different temperatures

**Fig. 6:**
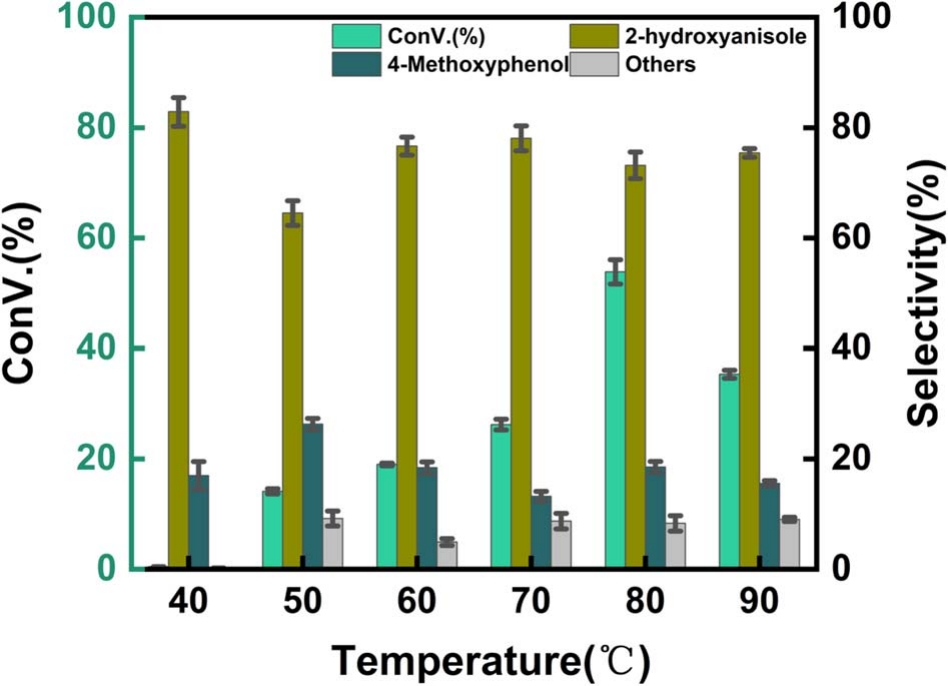
Effect of reaction temperature. Reaction conditions: anisole (90 mg), 7% Yb/CuY (1 mg), hydrogen peroxide (250 μL), solvent (5 mL acetonitrile and 5 mL water), 30 min.

The effect of reaction temperature on the hydroxylation of anisole was investigated in the range of 40–90 °C to determine the lowest temperature at which the highest conversion rate and optimal guaiacol selectivity could be achieved, as shown in Fig. 6. In reactions using hydrogen peroxide as the oxidant, temperature plays a critical role. Excessively high temperatures can lead to the decomposition of hydrogen peroxide into water and oxygen, causing unnecessary waste and reducing the conversion rate. Conversely, excessively low temperatures hinder the reaction from proceeding. From Fig. 6, the highest conversion rate, along with selectivity above 70%, was achieved at 80 °C. At temperatures above 80 °C, the conversion rate of anisole decreased, whereas temperatures below 80 °C resulted in a gradual increase in conversion rate with rising temperature. Increasing the temperature significantly enhanced the mass transfer rate and the reaction rate between anisole and hydroxyl radicals; however, excessively high temperatures led to hydrogen peroxide decomposition, ultimately reducing the conversion rate.

#### 3.2.3 Effect of solvent

**Fig. 7:**
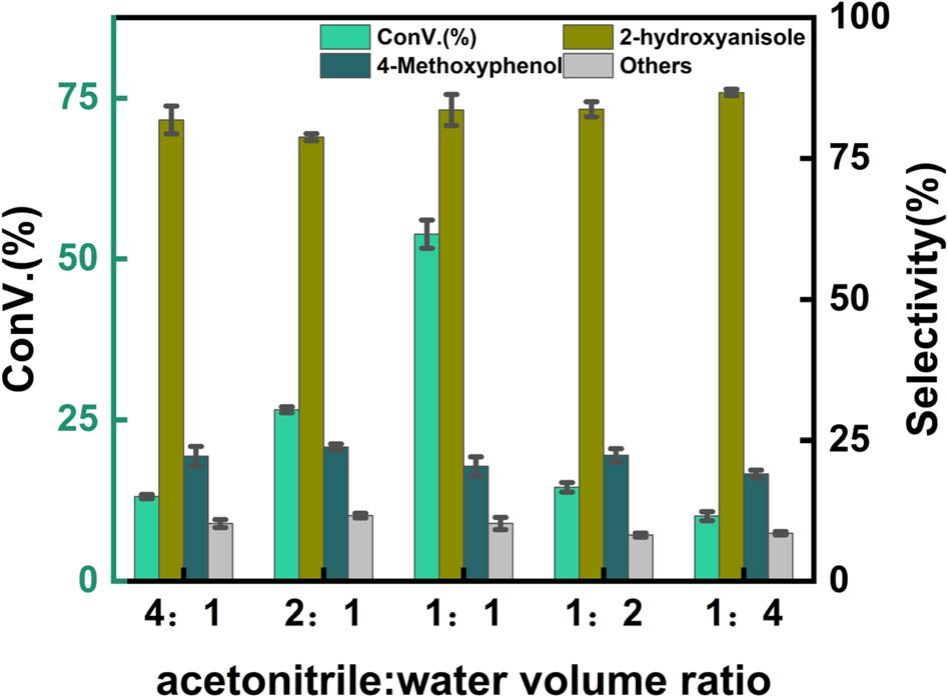
Effect of solvent. Reaction conditions: anisole (90 mg), solvent (10 mL), 7% Yb/CuY (1 mg), hydrogen peroxide (250 μL), 80 °C, 30 min.

Fig. 7 illustrates the effect of different volume ratios of acetonitrile to water on the reaction. The highest conversion rate was observed at a volume ratio of acetonitrile to water of 1 : 1. Additionally, when the volume ratio of acetonitrile to water exceeded 1 : 1, the conversion rate remained higher compared to ratios below 1 : 1. This is primarily because a higher proportion of acetonitrile enhances the solubility of anisole, increasing its reaction concentration and thereby boosting the conversion rate. However, an excessively high proportion of acetonitrile may reduce the effective concentration of hydrogen peroxide or destabilize reaction intermediates, leading to their degradation or conversion into by-products, ultimately decreasing the reaction conversion rate (Table 2).

**Table 2:** Cu and Yb content of fresh and used catalysts from ICP

7% Yb/CuY	Fresh	Used
Cu	4.72%	4.63%
Yb	6.51%	6.45%

#### 3.2.4 Effect of different substrate ratios

**Fig. 8:**
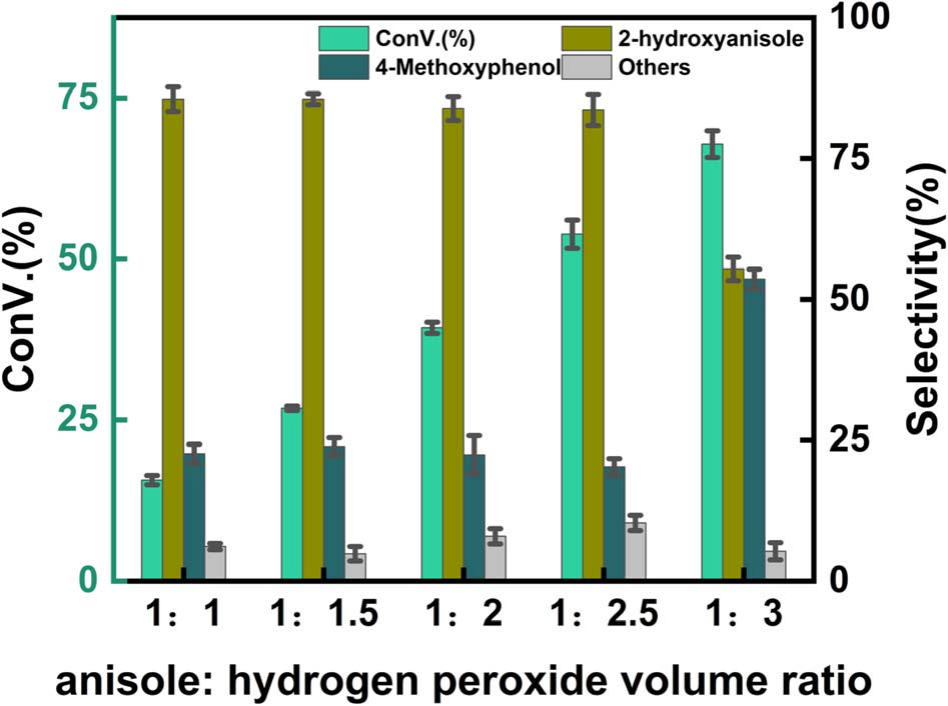
Effect of anisole to hydrogen peroxide volume ratio. Reaction conditions: anisole (90 mg), 7% Yb/CuY (1 mg), hydrogen peroxide (250 μL), solvent (5 mL acetonitrile and 5 mL water), 30 min.

Fig. 8 illustrates the effect of different anisole to hydrogen peroxide volume ratios on the reaction. The highest anisole conversion rate of 56.2% was achieved at an anisole to hydrogen peroxide volume ratio of 1 : 2.5, with guaiacol selectivity of 74.6%, resulting in a maximum guaiacol yield of 41.9%. As shown in Fig. 8, increasing the amount of hydrogen peroxide led to a gradual increase in anisole conversion, reaching its peak guaiacol yield at the 1 : 2.5 ratio. However, an excessive amount of hydrogen peroxide caused further oxidation of the product, reducing guaiacol selectivity.

#### 3.2.5 Recycling run and versatility performance

**Fig. 9:**
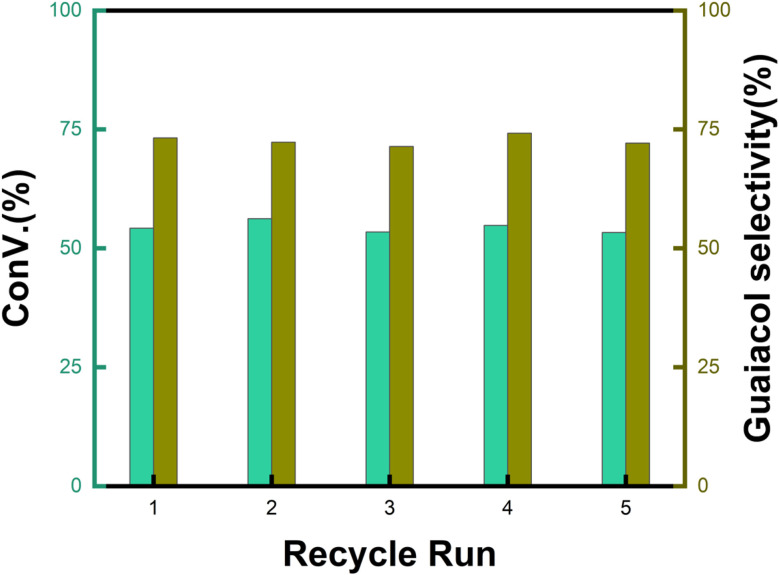
Recycling run performance of 7% Yb/CuY. Reaction conditions: anisole (90 mg), 7% Yb/CuY (1 mg), hydrogen peroxide (250 μL), solvent (5 mL acetonitrile and 5 mL water), reaction time: 30 minutes.

Fig. 9 shows the performance of the catalyst after being reused five times. After each use, the catalyst was recovered by centrifugation, washed with water and ethanol, dried, and calcined in a muffle furnace at 550 °C before reuse. The catalyst exhibited good stability across multiple reaction cycles, maintaining over 90% of its initial catalytic activity after five consecutive runs. This indicates that the structural integrity and oxidation states of the active components remained largely unchanged, demonstrating excellent reusability and resilience under the given reaction conditions. We performed ICP analysis before and after the reaction. Before the reaction, the Cu content in 7% Yb/CuY was 4.72%, and the Yb content was 6.51%. After the reaction, the Cu content was 4.63%, and the Yb content was 6.45%. The Cu and Yb contents remained almost unchanged before and after the reaction, indicating that the catalyst exhibits excellent stability and integrity. These results suggest that the catalyst can be effectively used for repeated applications without significant loss of performance. Additionally, the catalyst was evaluated for its versatility in catalyzing several anisole derivatives (Table 3), showing good catalytic activity for 4-fluoroanisole, 4-chloroanisole, and 4-bromoanisole.

**Table 3:** 7% Yb/CuY-catalyzed oxidation of anisole derivatives^a^

Entry	Reactant	ConV. (%)	
1	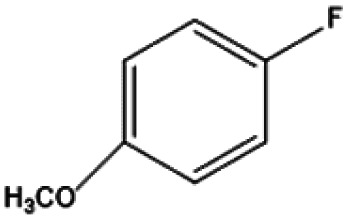	40.5%	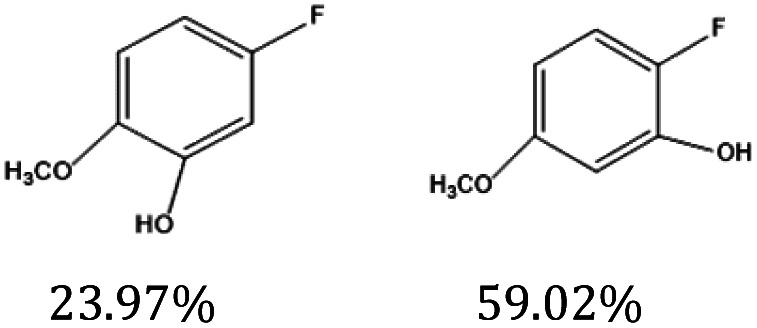
2	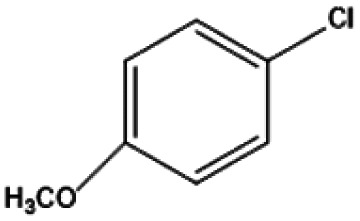	52.29%	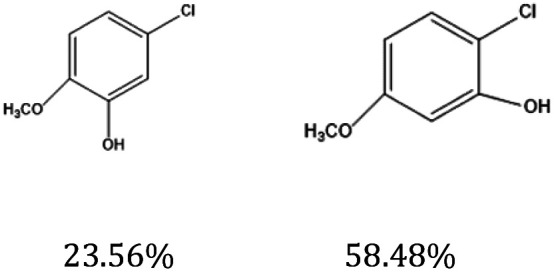
3	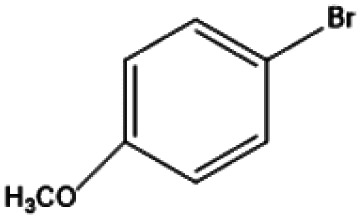	27.07%	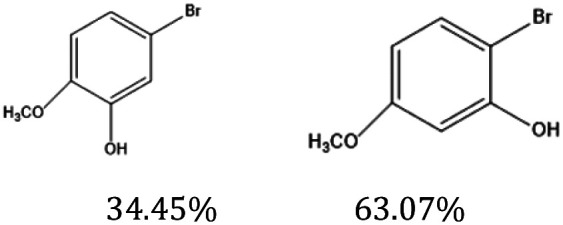

a Reaction conditions: 100 mg reactant, 7% Yb/CuY (1 mg), hydrogen peroxide (250 μL), solvent (5 mL acetonitrile and 5 mL water), reaction time: 30 minutes.

### 3.3 Reaction process

**Fig. 10:**
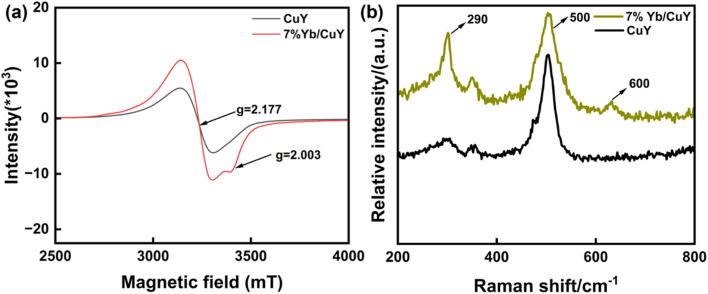
(a) Room-temperature ESR spectra (b) Raman spectra of CuY and 7% Yb/CuY.

As shown in Fig. 10(a), the ESR spectrum reveals that the *g*-value of 2.177 for CuY indicates that Cu^2+^ is predominantly located in the supercage of the NaY zeolite. In 7% Yb/CuY, an asymmetric peak at *g* = 2.003 appears, suggesting that the introduction of Yb creates oxygen vacancies in the zeolite, which is consistent with the XPS results. During the conversion of some Cu^2+^ to Cu^+^, oxygen vacancies are generated. These vacancies enhance the zeolite's ability to adsorb hydroxyl radicals, thereby increasing their utilization and preventing them from reacting with hydrogen peroxide to produce water and oxygen, which would otherwise lead to the waste of hydroxyl radicals.

Fig. 10(b) presents the Raman spectra of CuY and 7% Yb/CuY. The peak at 500 cm^−1^ is attributed to the vibration of oxygen atoms in NaY zeolite, perpendicular to the T–O–T bond (where T represents Si or Al). The peak at 290 cm^−1^ corresponds to Cu^+^–O vibrations. The introduction of Yb into CuY alters the electronic structure of Cu^2+^, converting some of it to Cu^+^. Additionally, a peak at 600 cm^−1^ appears in 7% Yb/CuY, compared to CuY, indicating the presence of oxygen vacancies. This suggests that the transformation of Cu^2+^ to Cu^+^ results in the loss of oxygen, leading to the formation of oxygen vacancies, which aligns with the EPR results.

**Fig. 11:**
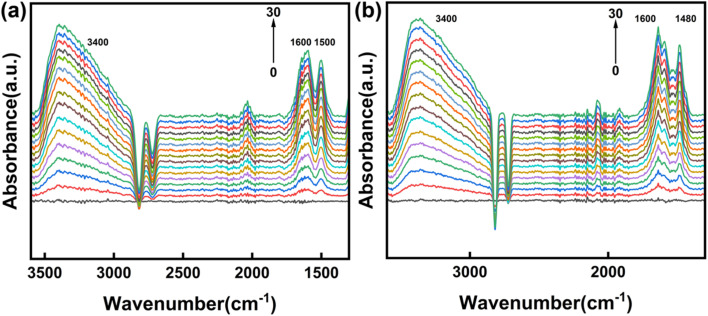
*In situ* DRIFTS of CuY (a), 7% Yb/CuY (b).

We collected *in situ* DRIFT spectra of the hydrogen peroxide oxidation of anisole over CuY and 7% Yb/CuY to investigate the reaction process. In the experiment, 1 mg of catalyst was used, with acetonitrile (5 mL) and water (5 mL) as solvents. Anisole (90 mg) and hydrogen peroxide (250 μL) were added, and the reaction was carried out at 80 °C for 30 minutes. The main peaks of all catalysts appeared in the 1400–1600 cm^−1^ range, corresponding to the hydroxyl-cyclohexadiene intermediate, a key species formed during anisole oxidation. Compared to CuY, 7% Yb/CuY exhibited stronger peak signals in this range, suggesting that Yb doping enhances the formation of active intermediates. Additionally, the peak of 7% Yb/CuY shifted from 1500 cm^−1^ to 1480 cm^−1^, indicating that Yb doping stabilizes the reaction intermediates. These effects contribute to a higher reaction conversion rate. The absorption peak at 3400 cm^−1^ is attributed to the vibration of the phenolic hydroxyl (–OH) group, indicating the formation of guaiacol (Fig. 11).

**Fig. 12:**
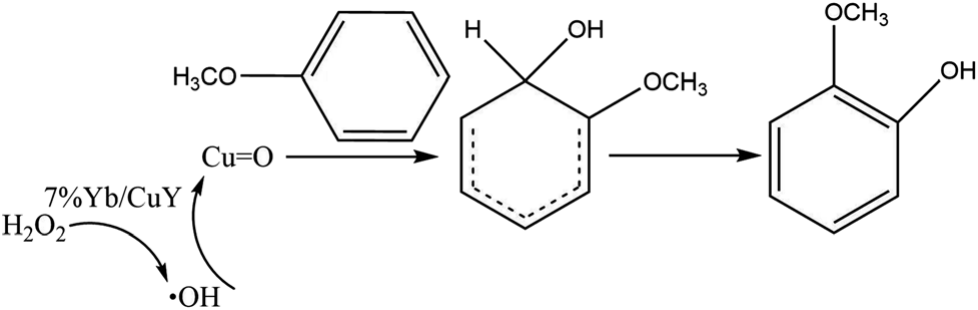
Probable catalytic mechanism.

Based on this, we propose the following reaction mechanism: 7% Yb/CuY catalyzes the decomposition of hydrogen peroxide to produce ˙OH radicals. These radicals then react with Cu to form a metal–oxygen intermediate. This intermediate subsequently attacks anisole, generating an active reaction intermediate that ultimately converts into guaiacol (Fig. 12).

## 4 Conclusion

In summary, we demonstrated that the Yb/CuY catalyst, prepared by introducing ytterbium species and a CuY zeolite, can significantly enhance the catalytic efficiency of hydrogen peroxide in oxidizing anisole to guaiacol. Characterization of the catalyst showed that the introduction of ytterbium increased the concentration of Cu^+^ species. By adjusting the loading amount of ytterbium, the ratio of Cu^2+^/Cu^+^ can be controlled, and an optimal ratio can fully exploit the synergistic effect of Cu^2+^/Cu^+^, effectively enhancing the conversion of hydrogen peroxide into hydroxyl radicals, thereby improving catalytic performance. The introduction of Yb creates oxygen vacancies in the catalyst, which improves its ability to adsorb hydroxyl radicals, promotes the formation of reaction intermediates, and increases the reaction conversion rate. In addition, the introduction of ytterbium effectively increased the number of strong acid sites on the CuY catalyst, which stabilized hydrogen peroxide and promoted electron transfer, thereby enhancing the utilization efficiency of hydrogen peroxide. This strategy could guide the design of efficient catalysts for aromatic hydroxylation with hydrogen peroxide and provide insights into Cu^+^ catalyst development.

## Data availability

The data supporting the results of this study are included within the manuscript. For any further data requests, please reach out to the corresponding author. Your inquiry will be promptly addressed to enhance the understanding of the research.

## Conflicts of interest

The authors declare no competing interests.

## Supplementary Material

RA-015-D5RA00463B-s001
