# Supplementary material for: Yb-Doped CuY modulates Cu electronic structures for efficient oxidation of anisole to guaiacol
Source: RSC Adv. 2025 Apr 1;15(13):9899–909. doi: 10.1039/d5ra00463b (PMC11959456; doi:10.1039/d5ra00463b)
Supplement: RA-015-D5RA00463B-s001 [file RA-015-D5RA00463B-s001.pdf]

Supplementary Information (SI) for RSC Advances.
This journal is © The Royal Society of Chemistry 2025

Supporting information

Table S1. Summary of BE of CuY、2%Yb/CuY、4%Yb/CuY and 7%Yb/CuY analyzed by XPS

Catalyst	BE of Cu $2p_{3/2}$(eV)		Cu^{2+}: Cu^{+}(at%)
	Cu^{2+}	Cu^{+}	
CuY	935.86eV		17.28:0
2%Yb/CuY	935.34eV	933.02eV	1.31:9.08
4%Yb/CuY	935.48eV	933.08eV	1.97:6.78
7%Yb/CuY	936.18eV	933.93eV	2.14:4.15

Table S2. NH3-TPD results of the NaY zeolites modified by various alkaline earth metals

Catalyst	Acidity amount(μmol/g)			Total acid amount (μmol/g)
	Weak	Medium	Strong	
CuY	555	952	0	1507
2%Yb/CuY	584	421	125	1140
4%Yb/CuY	627	456	128	1216
7%Yb/CuY	561	662	259	1482

Table S3. Surface areas and pore volumes of the modified NaY zeolites

Catalyst	A_{BET}/(m^2/g)	V_{micro}/(cm^3/g)
NaY	719.26	0.3322
CuY	697.12	0.3141
4%Yb/CuY	389.51	0.1930
7%Yb/CuY	279.68	0.1436

Table S4. The mass fractions of Yb and Cu by ICP-OES

	Yb(wt.%)	Cu(wt.%)
4%Yb/CuY	3.33%	5.70%
7%Yb/CuY	6.51%	4.72%
8%Yb/CuY	7.25%	3.95%

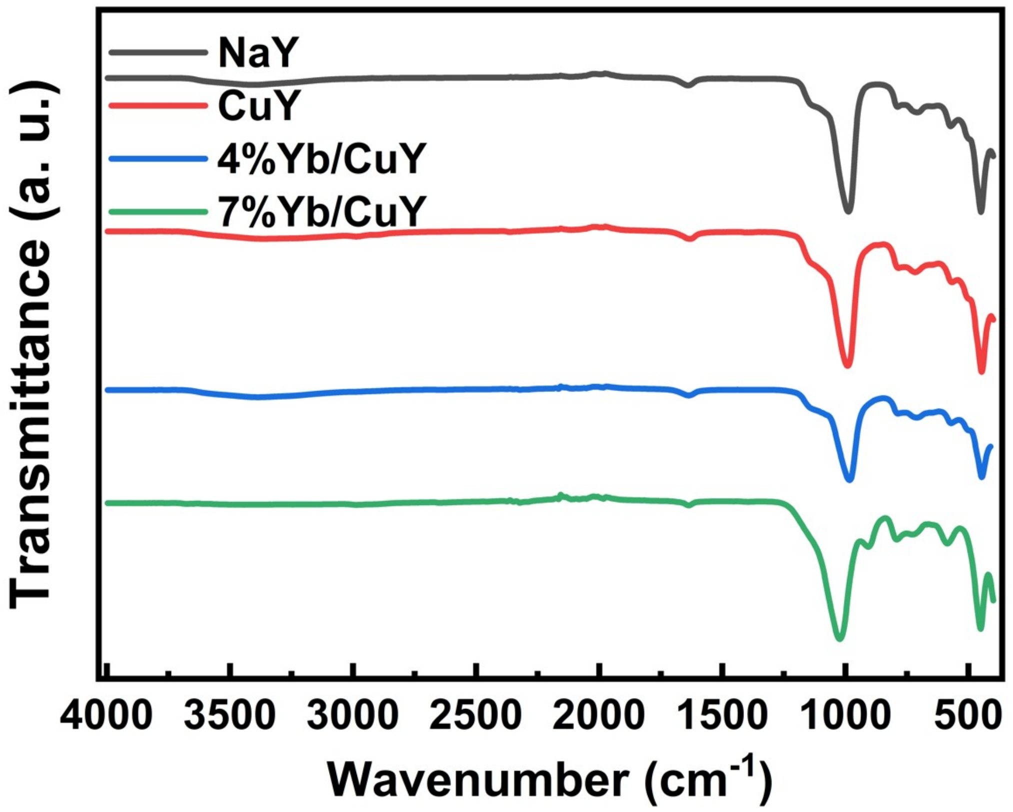

Figure S1. FTIR spectra of NaY, CuY, 4%Yb/CuY and 7%Yb/CuY

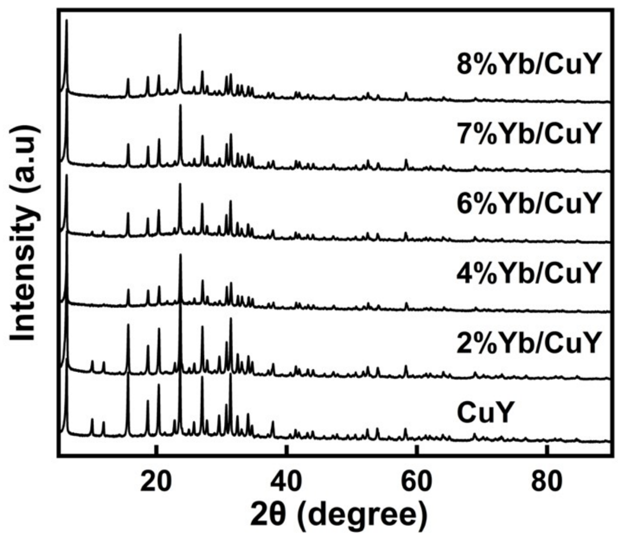

Figure S2. XRD patterns of CuY, 2% Yb/CuY, 4% Yb/CuY, 6% Yb/CuY， 7% Yb/CuY and 10% Yb/CuY.

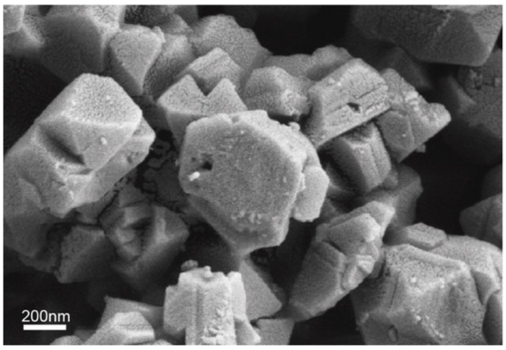

Figure S3. SEM image of 7% Yb/CuY

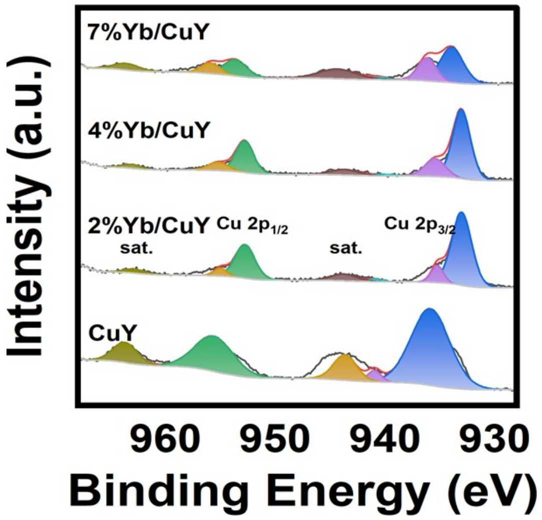

Figure S4. XPS spectra of Cu 2p of CuY, 2% Yb/CuY, 4% Yb/CuY and 7% Yb/CuY

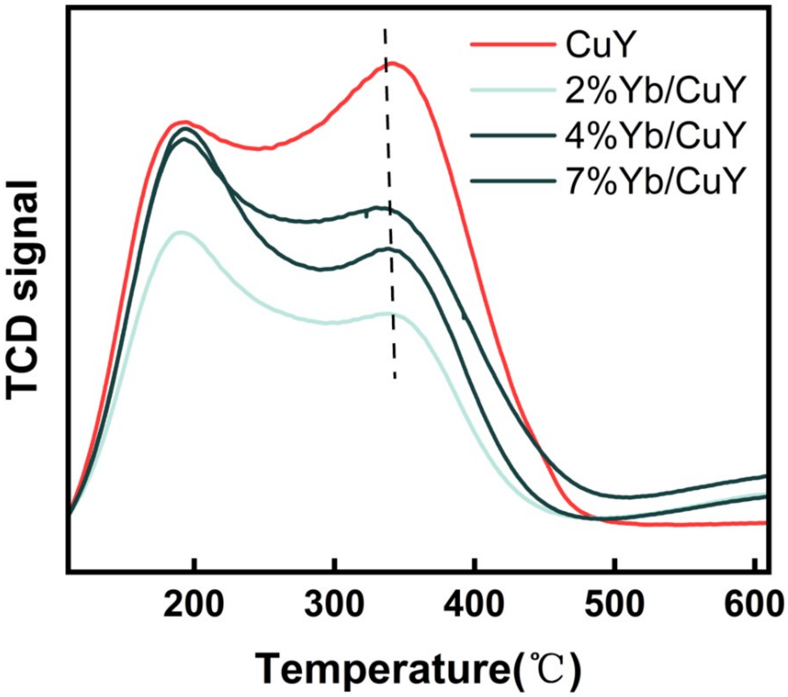

Figure S5. TPD of ammonia of CuY, 2%Yb/CuY, 4%Yb/CuY and 7%Yb/CuY.

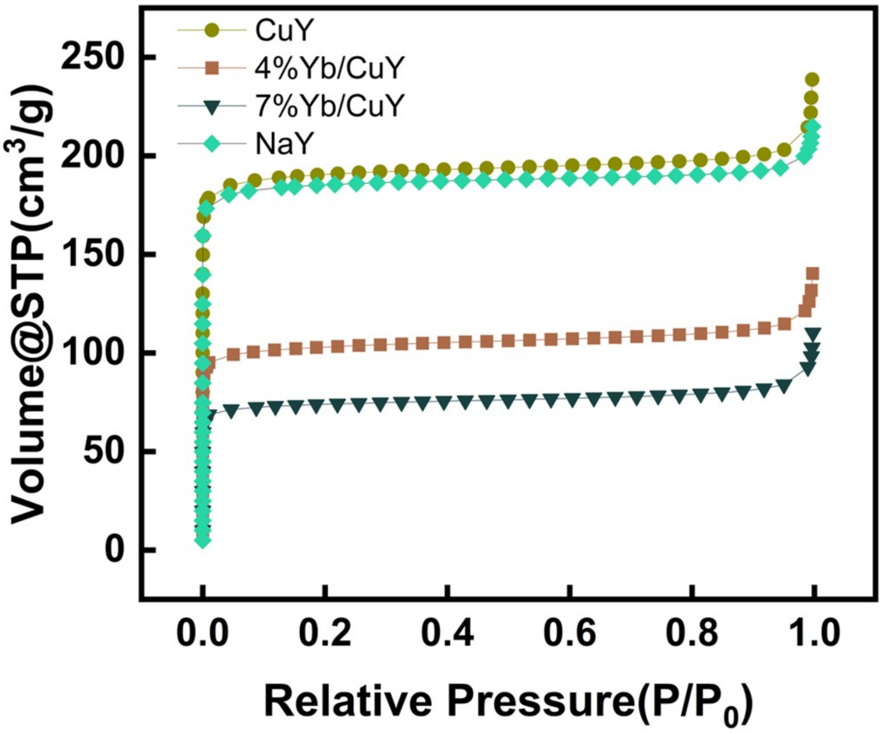

Figure S6. Nitrogen adsorption and desorption of NaY，CuY， 4%Yb/CuY and 7%Yb/CuY

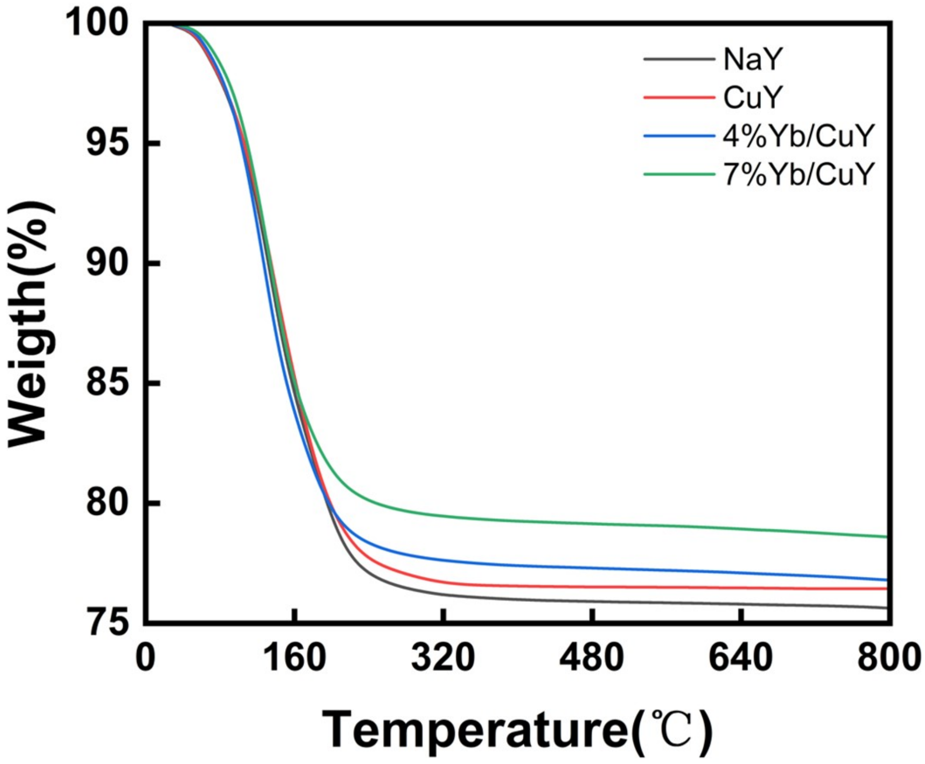

Figure S7. Desorption thermogravimetric (TG) curves of NaY，CuY， 4%Yb/CuY and 7%Yb/CuY
